# Alban Köhler, the pioneer of osteoradiology

**DOI:** 10.1007/s00264-026-06795-2

**Published:** 2026-03-28

**Authors:** Jan Bartoníček, Ondřej Naňka

**Affiliations:** 1 Department of Orthopedics, First Faculty of Medicine, Charles University and the Central Military Hospital, Prague, Czech Republic; 2 Institute of Anatomy, First Faculty of Medicine, Charles University, Prague, Czech Republic

**Keywords:** History of osteoradiology, Alban Köhler, Hip radiology, Teardrop

## Abstract

The terms Köhler´s teardrop, Köhler disease I and Köhler disease II are known to almost all orthopaedic surgeons and radiologists. However, little is known about this prominent personality of the German and world radiology, who described these structure or diseases. Alban Köhler (1874–1947) was one of the first German speaking pioneers in the emerging discipline – radiology, particularly radiology of bones and joints. He introduced new radiological techniques, such as “Teleröntgenographie”, and published a number of outstanding studies focused primarily on the musculoskeletal system, as well as on pulmonary tuberculosis and cardiac radiology. For a long time, Köhler had been actively involved in the study of radiation sickness and proposed measures that are valid to this day. The most famous Köhler´s publication “Lexikon der Grenzen des Normalen und der Anfänge des Pathologischen im Röntgenbilde” (The borderlands of the normal and early pathological in the skiagrams) was published for the first time in 1910. After Köhler´s death, the book was repeatedly published and gained recognition worldwide. Its 9th edition was initiated by Professor Emil Alfred Zimmer in 1953. Under a slightly modified title “Borderlands of normal and early pathological findings in skeletal radiology “, it is still published today, both in German and English.

## Introduction

The terms Köhler´s teardrop, Köhler disease I and Köhler disease II are known to almost all orthopaedic surgeons and radiologists. However, little is known about this prominent personality of the German and world radiology. His significance for the development of radiology has been mentioned only briefly in the German literature, most recently in 2024, 150 years after his birth [1–3]. The aim of this article is to provide a detailed overview of Köhler's personality and his work.

## Alban Köhler (1874 – 1947)

**Fig. 1:**
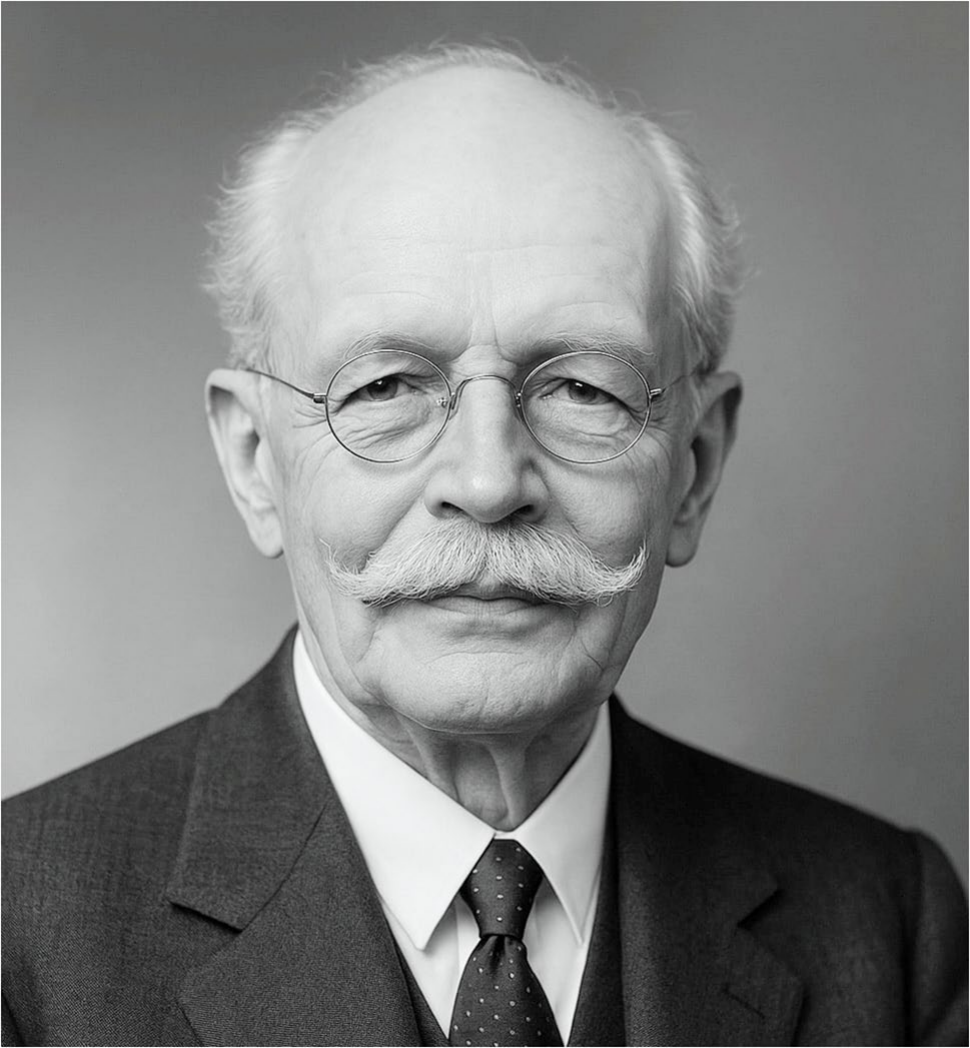
Professor Dr. Alban Köhler (1874–1947)

Alban Köhler (Fig. 1) was born on March 1, 1874, in the village of Petsa in Thuringia (Germany) into a farmer's family. As a child, he became enthusiastic about painting and various technical disciplines, including photography. He later made good use of the skills he had acquired, in his work as a radiologist. After graduating from high school in 1893, he began his university studies in Freiburg, continued in Leipzig and Erlangen, and completed them in Berlin in 1897. The topic of his dissertation thesis was pituitary tumours. The following year (1998), he joined the renowned German pathologist *Christian Georg Schmorl (1861–1932)* in Dresden*.* In 1899, he moved to Wiesbaden to St. Josef's Hospital to work in the surgical clinic of *Friedrich Cramer (1847–1903),* whose name was made famous by the Cramer's splint. Here he decided to devote himself to radiology and combined two fields, radiology and surgery, in his person.

Köhler first heard about radiology during his sixth semester of medical school, in 1895. In April 1899, he performed his first radiological examination on a six-year-old girl with a lower leg injury. In 1902, he married Elisabeth Kleine-Krahn, with whom he had a son. In 1906, he opened his own radiology institute in Wiesbaden, where he worked until 1945. In 1913, he was appointed Professor h.c. in Wiesbaden.

In 1933, at 24th Congress of the German Radiological Society in Bremen, Köhler received the Hermann Rieder Medal. At the end of his life, he suffered two tragedies. On the night of February 2 to 3, 1945, his radiology institute was completely destroyed by bombing. At around the same time, his only son, also a doctor, was killed on the Eastern Front. Köhler died on February 26, 1947, in Niederelters and was buried in Wiesbaden.

## Köhler's professional and publishing activities

Together with *Robert Kienböck (1871–1953), Heinrich Ernst Albers-Schönberg (1865–1921)* and *Rudolf Grashey (1876–1950),* Köhler was one of the first prominent German-speaking specialists in the newly emerging field of radiology, particularly radiology of bones and joints.

In 1905, Köhler co-founded the German Radiological Society—*Deutsche Röntgengesellschaft*. In April 1912, he presided 8th Radiological Congress in Berlin.

Köhler was technically highly skilled and introduced some new radiological techniques [4–7]. In 1904, he focused on "Teleröntgenographie," which was originally introduced by Albers-Schönberg with the aim of improving the quality of X-ray images. With the same goal in mind, Köhler proposed the "Doppelplateverfahren" (double plate technique) in 1906. In 1909, he laid the foundations for Sievetherapy (also known as Grid therapy), an irradiation technique using a grid to irradiate deep-seated tissues by spatially fractionated kilo- and megavoltage X-rays [6, 7]. In 1926, he published a study on the history of x-ray tubes [8].

In 1903, Köhler also focused on radiation sickness. From 1905, he performed X-ray examinations when standing behind a lead-shielded wall. Despite this, he observed hyperkeratosis on his fingers and toes. Protection against radiation was not common at that time. In 1910, his friend Albers-Schönberg had to undergo amputation of his left hand, which had been severely damaged by X-rays. At the Radiology Congress in Baden-Baden in 1931, Köhler recommended that all colleagues who had been working in the field for 20–30 years undergo a blood count test. In 1934, at 4th International Radiology Congress in Zurich, he reported on extensive global research concerning *"…the subjective overall problems of radiologists who have been working with X-rays for at least 25 years*".

Köhler focused his activities in the field of radiology primarily on the musculoskeletal system, pulmonary tuberculosis, and cardiac radiology [9–11]. He believed that X-rays were only part of the diagnostic process and that X-ray findings had to be compared with the clinical picture (condition, findings).

Köhler was the author of numerous articles and textbooks. In 1901, he published his first book, more precisely an atlas “Knochenerkrankungen im Röntgenbilde” (*Bone diseases in X-ray images*) [9] and in 1905 a textbook “Die normale und pathologische Anatomie des Hüftgelenkes und Oberschenkels in röntgenographischer Darstellung “ (*The normal and pathological anatomy of the hip joint and thigh in radiographs*) [10], which was apparently the first monograph devoted to the hip joint. In 1906, he published a book “Zur Röntgendiagnostik der kindlichen Lungendrüsentuberkulose” (*On X-ray diagnosis of pulmonary tuberculosis in children*) [11] and in 1910 there appeared the first edition of “Lexikon der Grenzen des Normalen und der Anfänge des Pathologischen im Röntgenbilde” (*Encyclopedia of the limits of normality and the beginnings of pathology in X-ray images*), which over the years made Köhler famous throughout the world (see below) [12].

## Köhler´s eponyms and bone terminology

Several bone structures and disorders are associated with Köhler's name, which he described himself or contributed to their description.

### Köhler´s teardrop

**Fig. 2:**
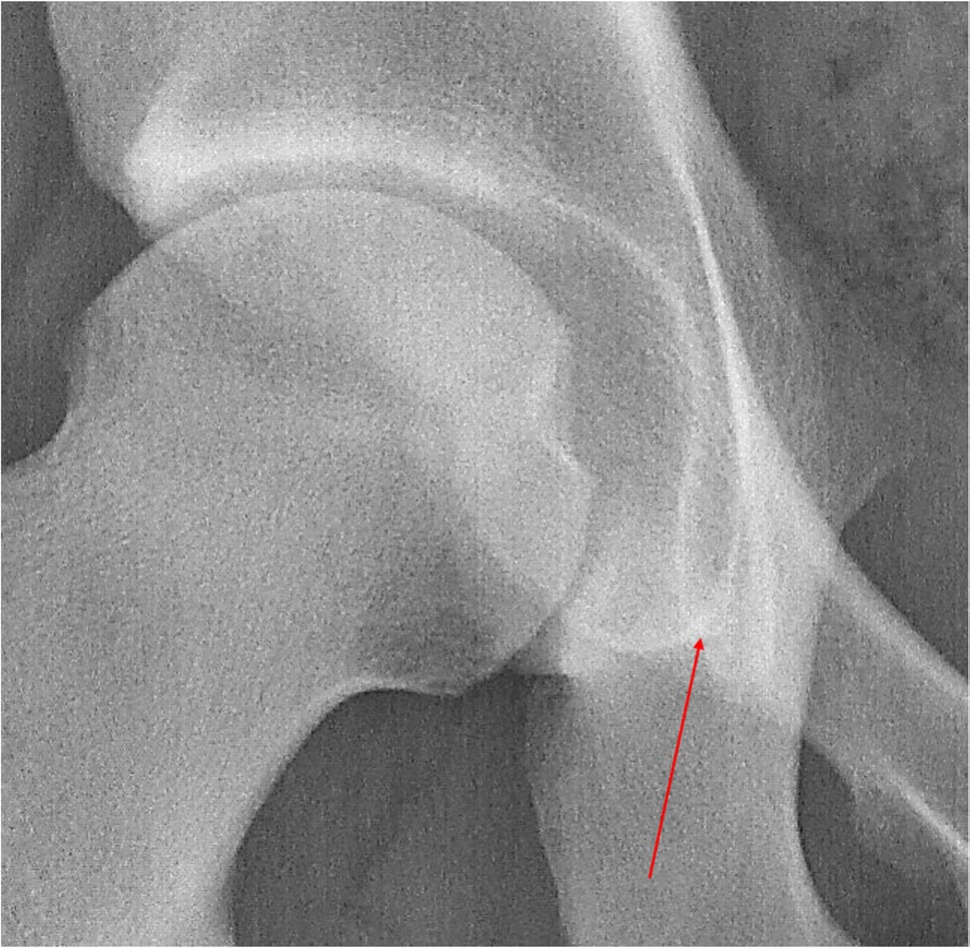
Radiograph of the right hip joint – a detail showing the “Köhler teardrop” (red arrow)

**Fig. 3:**
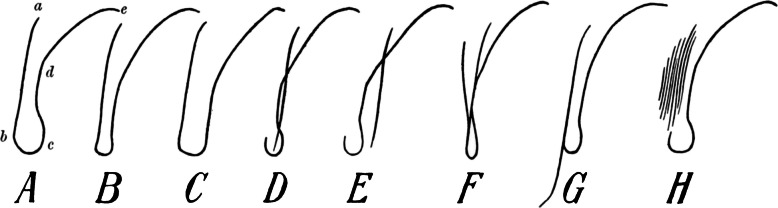
Original Köhler drawings depicting various shapes of the teardrop [10]

**Fig. 4:**
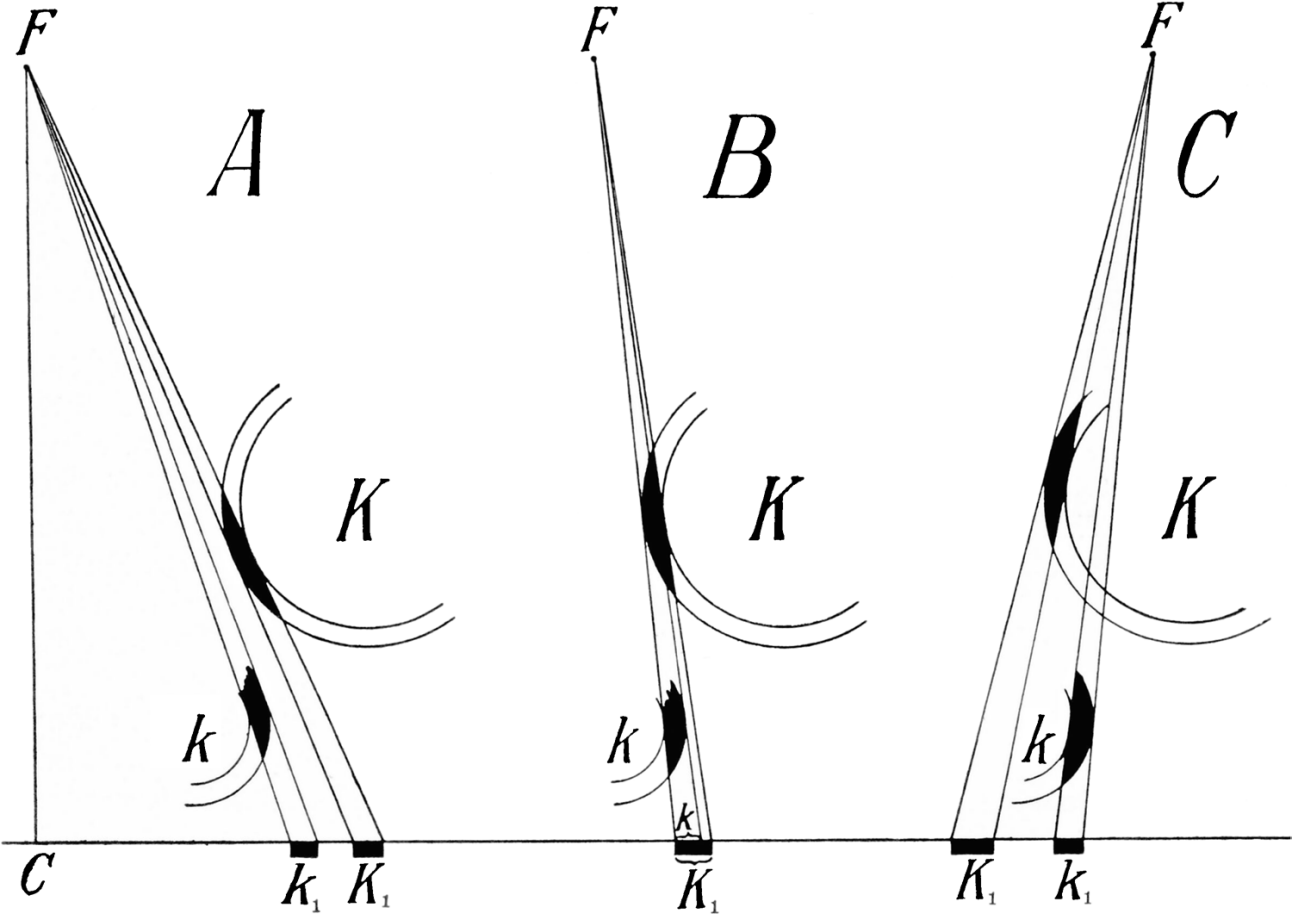
Köhler's original drawings showing the change in the shape of the teardrop depending on the direction of the X-ray beams [10]

The teardrop-shaped structure between the inferomedial part of the acetabulum and the ilioischial line on a pelvic radiograph (Fig. 2) is referred to in the literature as “Köhler's tear”, “die Köhlersche Tränenfigur”, “teardrop “ or “U-figure “ [13–19]. Köhler described it for the first time in 1905 in the book “Die normale und pathologische Anatomie des Hüftgelenkes und Oberschenkels in röntgenographischer Darstellung” (*The normal and pathological anatomy of the hip joint and thigh in radiographs*), apparently the first monograph dealing with radiology of the hip joint [10]. Here, the author examined with incredible thoroughness and inventiveness the formation of a teardrop-shaped shadow, which, in his view, was created by the superimposition of the inner surface of the acetabulum, the acetabular notch, and the posterior horn of the facies lunata. He concentrated on the influence of the patients' age (growing versus mature skeleton), the position of the X-ray tube, the tilt of the pelvis, and a number of other details (Fig. 3 and 4). Köhler named the formation studied “*das Wassertropfen “* (water drop) or *“die Glasthräne* “. The description of the teardrop subsequently appeared also in the book “*Grenzen des Normalen und Anfänge des Pathologischen im Röntgenbilde “* ([12]. As a result, this structure has been given the name “*die Köhlersche Tränenfigur*” in the German literature, while in the English literature the term “*teardrop*” has become established [13–19].

The anatomy of Köhler's tear has become a subject of a number of studies [16, 17]. According to them, this structure is formed by the summation of several pelvic structures, namely the cortex in the anterior part of the acetabular fossa (lateral border), the cortex of the medial wall of the pelvis in the posterior part of the fossa (medial border), and the horizontal part of the line delimiting the anterior part of the obturator foramen (distal border). In radiological practice, Köhler's tear is an important landmark for assessing the shape and integrity of the acetabulum. In 1961, Sharp [13] used this structure in the construction of an angle for assessing the inclination of the acetabulum. In 1964, the Judet brothers and Letournel [14] used the term *"U structure"* in a study devoted to acetabular fractures to evaluate the type of fracture. In 1969, Hubbard [15] used the teardrop and the so-called Köhler line to evaluate acetabular protrusion.

### Köhler-Pellegrini-Stieda disease

This term is used to refer to ossified post-traumatic lesions at (or close to) the medial collateral ligament adjacent to the border of the medial femoral condyle.

Alban Köhler reported in 1905 a case of a 56-year-old male who injured his knee during piling of wood in 1903 [10]. The Italian orthopedist *Augusto Pellegrini (1877–1958)* described this condition in the same year based on a case from 1904 [20]. The German surgeon *Alfred Stieda (1869–1945)* reported on the same entity in 1908 [21], without noting the descriptions of his predecessors.

### Köhler disease I

In 1908, Köhler described "osteonecrosis" of the navicular bone in three paediatric patients aged five to nine years [22]. He mentioned the course of the disease and discussed in detail its possible aetiology, including differential diagnosis. Köhler did not use the term necrosis at that time, but wrote literally about "isolated crippling or disfigurement" (isolierte Verkrüppelung).

### Freiberg-Köhler disease [Köhler disease II]

**Fig. 5:**
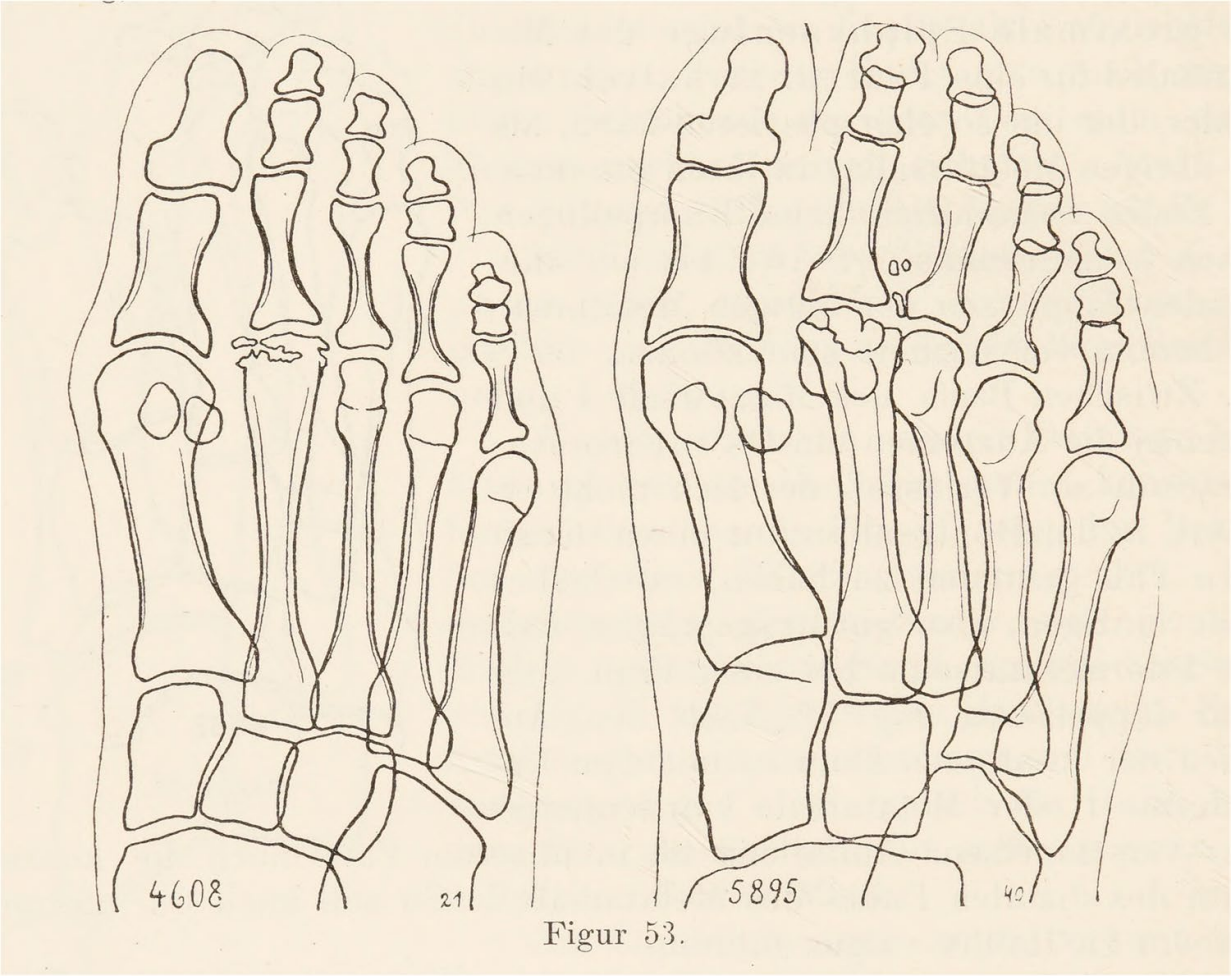
Köhler's original drawings of flattening of the joint surface of the head of the second metatarsal bone [25]

Osteochondrosis of the metatarsal heads (typically 2nd metatarsal head) is characterized by subchondral bone collapse, osteonecrosis, and cartilaginous fissures. The first to describe this entity was an American orthopedist *Albert Henry Freiberg (1868 – 1940)* in 1914 and called it “infraction of the second metatarsal bone “ [23]. Köhler described this disease briefly in 1915 [24] and in greater detail in 1920 [25], (Fig. 5). as Abflachung (flattening of the joint surface of the head of the second metatarsal bone). Freiberg returned to this issue in 1926 [26], when, among other things, he discussed the Köhler's description and his views on the etiology of the disease in his article.

### Metaphysis

Köhler was one of the first authors to start using, in 1905, [10] the new term “metaphysis” for the part of the bone between the diaphysis and the epiphysis [27]. This term was introduced in 1896 by a Swiss surgeon *Theodor Kocher (1841–1917)* based on his study of gunshot wounds to bones [28, 29]. Thanks to Köhler, this term began to be used in radiology before it appeared in anatomy textbooks [30].

## The borderlands of the normal and early pathological in the skiagrams

This book, which is the Köhler's most important work, was first published in 1910 under the title “*Lexikon der Grenzen des Normalen und der Anfänge des Pathologischen im Röntgenbilde “* [12]. It was based on an analysis of more than 10,000 X-ray images and the radiological literature available at the time. The second edition in 1915 [24] was already titled “*Grenzen des Normalen und Anfänge des Pathologischen im Röntgenbilde “*. The first English edition “*Röntgenology – The borderlands of the normal and early pathological in the skiagrams “* appeared in 1928 [31] and was followed by the French edition in 1930, the Spanish edition in 1933 and the Italian edition in 1955.

After Köhler's death, *Prof. Emil Alfred Zimmer (1908–1981)* took over, in 1953, 9th German edition of the book [32], which was already recognized worldwide at the time. The title of the book was changed to “Grenzen des Normalen und Anfänge des Pathologischen im Röntgenbilde des Skeletts “ (*Borderlands of normal and early pathological findings in skeletal radiology*). Zimmer also edited the subsequent 10th and 11th editions. After his death, the books were taken over by the next generation of editors. The latest 14th German edition was published in 2001 [33] and the latest 5th English edition in 2003 [34].

**Fig. 6:**
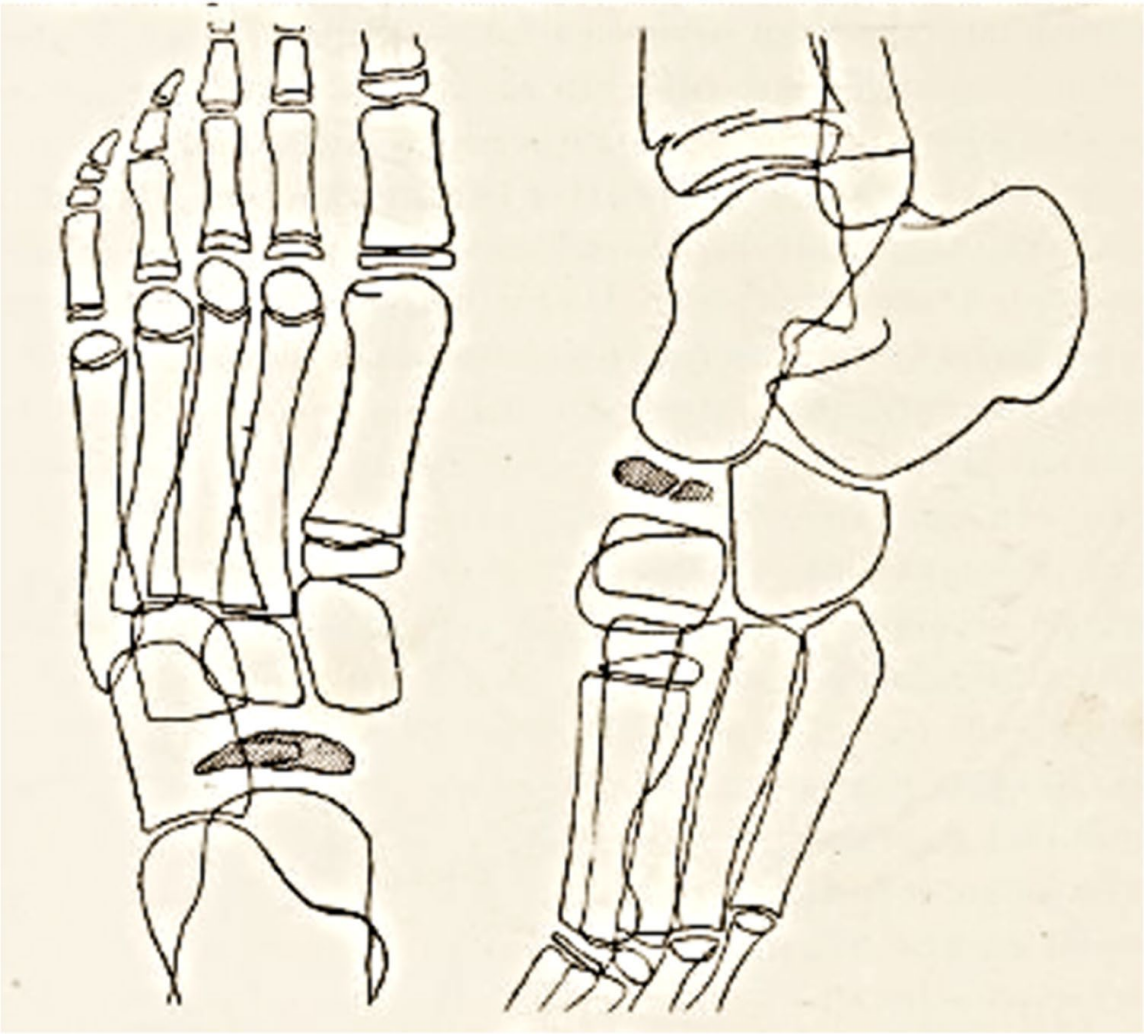
Köhler's original drawings of "isolated crippling or disfigurement" of the navicular bone [24]

Ninety years passed between the first and last editions. The first edition had 177 pages and contained 73 drawings by Köhler himself (Fig. 6), with 70% of the content devoted to the skeleton. The eighth edition, published in 1943, had 900 pages. The last English edition exceeded 1,100 pages and contained 3,000 figures. It is remarkable that Köhler's original drawings were used in the book long after his death. Since its inception, this book has been and continues to be one of the cornerstones of bone radiology literature.

## Conclusion

The above overview shows that the Köhler's achievements not only affected bone radiology, but also improved radiological diagnostics, radiotherapy and protection against radiation sickness. Köhler's work thus ranked him among the most important radiologists of his time.

## Data Availability

No datasets were generated or analysed during the current study.

## Declarations

### Clinical trial

Not applicable, it is a historic study.

## References

[1] Freyschmidt J (1995) Alban Köhler, ge. 1874, gest. 1947. Beurteilung eines Pioniers der klinischen Radiologie aus heutiger Sicht. Fortschr Röngenstr 163:463–468 (German) 8547614

[2] Laissue JA, Blattmann H, Slatkin DN (2012) Alban Köhler (1874-1947): Erfinder der Gittertherapie. Z Med Phys 22(2):90–99. 10.1016/j.zemedi.2011.07.002 21862299 10.1016/j.zemedi.2011.07.002

[3] Stuhrmann M (2024) Alban Köhler – Ein Rückblick zu seinem 150. Geburtstag anhand seiner „Lebenserinnerungen“ und seiner Röntgenbücher. Rofo 196(9):905–911 (German) 38626883 10.1055/a-2284-5540

[4] Köhler A (1905) Röntgenröhre mit Vorrichtung zur therapeutischen Dosierung der Röntgenstrahlen. Munch Med Wschr 2:76–77

[5] Köhler A (1909) Zur Röntgentiefentherapie mit Massendosen. Munch Med Wochenschr 56:2314–2316

[6] Köhler A (1909) Theorie einer Methode, bisher unmöglich unanwendbar hohe Dosen Röntgenstrahlen in der Tiefe des Gewebes zur therapeutischen Wirksamkeit zu bringen ohne schwere Schädigung des Patienten, zugleich eine Methode des Schutzes gegen Röntgenverbrennung überhaupt. Fortschr Geb Roentgenstr 14:27–29 (German)

[7] Köhler A (1909) A method of deep roentgen irradiation without injury to the skin. Arch Roentgen Ray 14(5):141–142

[8] Köhler A (1926) Über historische Rontgenrohren 1896-1898. Acta Radiol 7:1–6. 10.3109/00016922609138255

[9] Köhler A (1901) Knochenerkrankugen im Röntgenbile. Bergmann, Wiesbaden

[10] Köhler A (1905) Die normale und pathologische Anatomie des Hüftgelenkes und Oberschenkels in röntgenographischer Darstellung. Gräfe & Sillem, Hamburg

[11] Köhler A (1906) Zur Röntgendiagnostik der kindlichen Lungendrüsentuberkulose. Gräfe & Sillem, Hamburg

[12] Köhler A (1910) Lexikon der Grenzen des Normalen und der Anfänge des Pathologischen im Röntgenbilde. Gräfe & Sillem, Hamburg

[13] Sharp IK (1961) Acetabular dysplasia – the acetabular angle. J Bone Joint Surg 43-B:268–272

[14] Judet R, Judet J, Letournel E (1964) Fractures of acetabulum: classification and surgical approaches for open reduction. J Bone Joint Surg 46-A:1615–1675 14239854

[15] Hubbard MJS (1969) The measurement of progression in protrusio acetabuli. AJR Am J Roentgenol 106:506–508. 10.2214/ajr.106.3.506 10.2214/ajr.106.3.506 5794023

[16] Kölbel R, Golzo H (1977) Die Köhlersche Tränenfigur. RöFo - Fortschritte auf dem Gebiet der Röntgenstrahlen und der bildgebenden Verfahren 127:326–333. 10.1055/s-0029-1230710 10.1055/s-0029-1230710 144652

[17] Bowerman JW, Sena JM, Chang R (1982) The teardrop shadow of the pelvis; anatomy and clinical significance. Radiology 143:659–662. 10.1148/radiology.143.3.7079492 7079492 10.1148/radiology.143.3.7079492

[18] Sweeney JP, Helms CA, Minagi H, Louie KW (1987) The widened teardrop distance: a plain film indicator of hip joint effusion in adults. AJR Am J Roentgenol 149(1):117–119. 10.2214/ajr.149.1.117 3495970 10.2214/ajr.149.1.117

[19] Heinz K, Nowack D, von Eisenhart-Rothe R, Wassilew G, Matziolis G, Brodt S (2023) Koehlers teardrop is not a reliable landmark for assessing the centre of rotation after total hip arthroplasty. Arch Orthop Trauma Surg 143(9):5671–5676. 10.1007/s00402-023-04859-1 37099164 10.1007/s00402-023-04859-1 PMC10449955

[20] Pellegrini A (1905) Ossificazione traumatica del ligamento collaterale tibiale dell’articolazione del ginocchio sinistro. Clinica moderna (Firenze) 11:433–439

[21] Stieda A (1908) Uber eine typische Verletzung am unteren Femurende. Arch Klin Chir 85:815–826

[22] Köhler A (1908) Ueber eine häufige, bisher anscheinend unbekannte Erkrankung einzelner kindlicher Knochen. Münchn Med Wschr 55:1923–1925

[23] Freiberg AH (1914) Infraction of the second metatarsal – a typical injury. Trans South Surg Gynecol Assoc 26:171–174

[24] Köhler A (1915) Grenzen des Normalen und Anfänge des Pathologischen im Röntgenbilde, 2. Auflage. Lucas Gräfe & Sillem, Hamburg

[25] Köhler A (1920) Eine typische Erkrankung des zweiten Metatarsophalangealgelenkes. Munch Med Wochenschr 67:1289–1290

[26] Freiberg AH (1926) The so called offense of the second metatarsal bone. J Bone Joint Surg 8:257–261

[27] Naňka O, Bartoníček J (2024) Terminology of the growing bone: a historical study. Clin Anat 37(7):761–768. 10.1002/ca.24176 38778675 10.1002/ca.24176

[28] Kocher T (1895) Zur Lehre von den Schusswunden durch Kleinkalibergeschosse. Th. G. Fisher, Cassel

[29] Kocher T, Tavel E (1895) Vorlesungen über chirurgischen Infektionskrankheiten. Carl Sallmann, Basel-Leipzig

[30] Köhler A (1896) Bücherschau. Kocher, Zur Lehre von den Schusswunden durch Kleinkalibergeschosse. Dtsch Med Wochenschr 22:73

[31] Köhler A (1928) Röntenology – The borderlands of the normal and early pathological in the skiagrams. (translation of 5the German Edition) New York, William Wood

[32] Köhler A, Zimmer EA (1953) Grenzen des Normalen und der Anfänge des Pathologischen im Röntgenbild des Skellets, 9. Aufglage. Thieme, Stuttgart

[33] Brossman J, Czerny C, Freyschmidt J (2000) Freyschmidt´s Köhler/Zimmer: Grenzen des Normalen und Anfänge des Pathologischen, 14. Auflage. Thieme, Stuttgart

[34] Brossman J, Czerny C, Freyschmidt J (2003) Koehler/Zimmer Borderlands of Normal and Early Pathological Findings in Skeletal Radiology, 5th edition. Thieme, Stuttgart

[35] Köhler A (1920) Grenzen des Normalen und Anfänge des Pathologischen im Röntgenbilde, 3. Auflage. Lucas Gräfe & Sillem, Hamburg

